# Processing-Dependent Acrylamide Formation in French Fries: Optimisation of QuEChERS-Based Extraction and HPLC-PDA Determination

**DOI:** 10.3390/molecules31162777

**Published:** 2026-08-10

**Authors:** Nimo Hussein Yussuf, Eylem Odabas, Fatma Oznur Afacan, Bulent Kabak

**Affiliations:** 1 Department of Nutrition and Food Science, Edna Adan University, Iftin Road, Hargeisa, Somalia; 230474005@ogrenci.hitit.edu.tr; 2 Department of Food Engineering, Faculty of Engineering and Natural Sciences, Hitit University, Corum 19030, Türkiye; eylem.odabas@ogrenci.hitit.edu.tr; 3 Department of Nutrition and Dietetics, Hamidiye Faculty of Health Sciences, University of Health Sciences, Istanbul 34668, Türkiye; 241002502@ogrenci.sbu.edu.tr

**Keywords:** acrylamide, thermal processing, Maillard reaction, QuEChERS, HPLC, method validation

## Abstract

Acrylamide is a thermally induced process contaminant formed predominantly through Maillard-type reactions in carbohydrate-rich foods and is considered a major food safety concern due to its potential genotoxic and carcinogenic properties. In the present study, a modified QuEChERS (quick, easy, cheap, effective, rugged, and safe)-based extraction and clean-up procedure followed by high-performance liquid chromatography coupled with photodiode array detection (HPLC-PDA) analysis was developed and optimised for the determination of acrylamide in French fries. Different clean-up strategies involving primary secondary amine (PSA), C18, and salt-assisted extraction systems were comparatively evaluated to improve analytical selectivity and chromatographic performance in complex potato matrices. The optimised procedure demonstrated satisfactory analytical performance and chromatographic selectivity for acrylamide analysis in thermally processed potato matrices, with an LOQ value of 32.3 µg kg^−1^, recoveries ranging from 79.4 to 86.6%, and relative standard deviation (RSD) values below 8%. The validated method was subsequently applied to investigate the influence of thermal processing conditions on acrylamide formation in frozen French fries processed using sunflower oil, riviera olive oil, and palm oil. Deep-fat frying formed substantially higher acrylamide concentrations (526.7–829.6 µg kg^−1^) than oven baking (285.5–480.6 µg kg^−1^) and air frying (272.1–673.6 µg kg^−1^), whereas repeated frying cycles markedly enhanced acrylamide formation, particularly in sunflower oil systems. The proposed analytical strategy offers a reliable and analytically robust approach for acrylamide determination while providing mechanistic insights into processing-dependent chemical transformations associated with acrylamide formation in potato-based foods.

## 1. Introduction

Food quality and safety increasingly depend on the identification and control of chemically induced hazards formed during food processing. Among these hazards, thermally induced process contaminants have attracted substantial scientific and regulatory attention due to their toxicological significance and widespread occurrence in processed foods. In particular, acrylamide has emerged as one of the most intensively investigated heat-induced contaminants in carbohydrate-rich foods because of its potential genotoxic and carcinogenic properties [1]. Since its first identification in thermally processed foods in 2002 [2], acrylamide has become a major concern in food chemistry, prompting extensive research into its formation mechanisms, analytical determination, and mitigation strategies.

**Figure 1:**
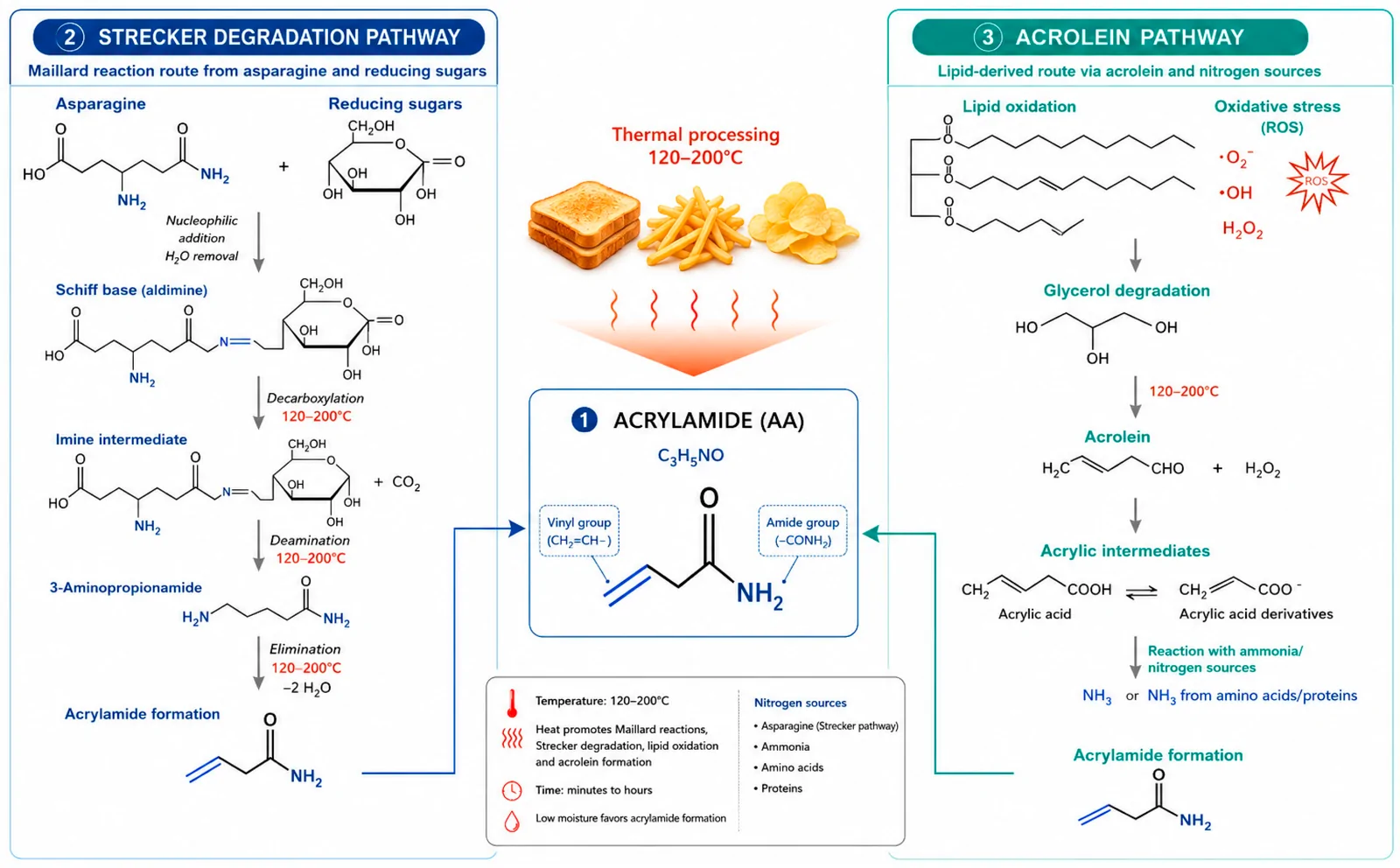
Proposed chemical pathways involved in acrylamide formation during thermal processing of potato-based foods, including Maillard reaction-mediated Strecker degradation and lipid-derived acrolein pathways.

Acrylamide is a highly polar, low-molecular-weight vinyl compound predominantly formed during high-temperature processing (>120 °C) of carbohydrate-rich foods through complex thermally induced reactions [1,3]. Acrylamide formation is primarily associated with Maillard-type reactions involving free asparagine and reducing sugars. During thermal processing, decarboxylation and deamination reactions generate reactive intermediates that contribute to acrylamide formation via Strecker-type degradation pathways [4]. In addition, alternative pathways involving acrolein intermediates formed during lipid thermolysis have also been proposed [5]. Acrylamide formation is therefore strongly influenced by precursor composition, water activity, pH, processing temperature, heating duration, and lipid environment. The principal chemical pathways associated with acrylamide formation during thermal processing are summarised in Figure 1.

Potato-based products are recognised as major contributors to dietary acrylamide exposure due to their high concentrations of reducing sugars and free asparagine combined with intensive thermal processing conditions [1]. French fries, in particular, constitute a critical food matrix in which acrylamide formation is highly dependent on processing variables such as frying technology, oil composition, thermal stability of the frying medium, repeated oil usage, and pre-treatment applications. Variations in heat transfer, dehydration kinetics, and oil–matrix interactions during deep-fat frying, oven baking, and air frying may substantially alter acrylamide-forming reaction pathways. Similarly, pre-treatment applications such as soaking and salt addition may influence precursor availability and reaction kinetics through diffusion-driven chemical modifications within potato tissues [6,7,8,9,10,11]. Due to its toxicological relevance, acrylamide has been classified as a Group 2A “probable human carcinogen” by the International Agency for Research on Cancer [12], while the European Commission has established benchmark levels for several potato-based products, including French fries (500 µg kg^−1^) and potato crisps (750 µg kg^−1^), to encourage mitigation practices during food processing [13].

The reliable determination of acrylamide in complex food matrices remains analytically challenging because of its high polarity, low molecular weight, limited chromophoric properties, and susceptibility to matrix interference [1]. Therefore, considerable efforts have focused on the development of robust analytical methodologies combining efficient sample preparation with selective chromatographic determination. Instrumental techniques based on gas chromatography (GC) [14,15,16], high-performance liquid chromatography coupled with photodiode array detection (HPLC-PDA) [17,18,19], and liquid chromatography-tandem mass spectrometry (LC-MS/MS) [7,10,20,21] have been widely applied for acrylamide analysis. However, GC-based approaches generally require derivatisation procedures due to the low volatility and polar nature of acrylamide, thereby increasing analytical complexity and extending sample preparation time [22]. Although liquid chromatographic techniques provide important advantages for the determination of polar and non-volatile analytes [23], French fries and related potato products remain analytically challenging matrices because their elevated starch and lipid contents may induce significant matrix effects, including signal suppression or enhancement [24].

For this reason, optimisation of extraction and clean-up procedures represents a critical aspect of analytical method development for acrylamide determination. Various methodologies, including liquid–liquid extraction, accelerated solvent extraction, and solid-phase extraction (SPE), have been proposed to minimise matrix interference and improve analytical sensitivity. However, many of these approaches involve laborious procedures, extensive solvent consumption, and multiple purification stages [25]. More recently, QuEChERS (quick, easy, cheap, effective, rugged, and safe)-based methodologies have attracted increasing attention as simplified and cost-effective alternatives offering satisfactory analytical performance for complex food matrices [26,27]. Furthermore, additional clean-up procedures involving lipid removal and Carrez-based precipitation of proteins and colloidal compounds have been reported to improve analytical reliability in starch- and lipid-rich food systems [26,28].

Although substantial has been made progress in elucidating the mechanisms of acrylamide formation and developing analytical methods for its determination, important knowledge gaps remain. Previous studies have predominantly focused on either analytical method development or individual processing factors affecting acrylamide formation, with comparatively limited attention devoted to integrating these aspects within a single experimental framework. Studies providing an integrated evaluation of analytical methodology and processing-dependent acrylamide formation in French fries remain limited. To address these knowledge gaps, the present study aimed to develop and optimise modified QuEChERS-based extraction and clean-up strategies for the determination of acrylamide in French fries using HPLC-PDA analysis and to comparatively evaluate the analytical performance characteristics of the proposed methodology. Furthermore, the optimised analytical approach was applied to investigate the influence of different thermal processing techniques, including deep-fat frying, oven baking, and air frying, together with different frying oils and pre-treatment applications, on acrylamide formation in frozen French fries. Particular emphasis was placed on processing-dependent chemical transformations affecting acrylamide formation in potato-based food systems under different thermal processing conditions.

## 2. Results and Discussion

### 2.1. Optimisation of Extraction and Clean-Up Procedure

The extraction and clean-up procedure for acrylamide determination was optimised in order to improve chromatographic selectivity and minimise matrix-derived interferences associated with thermally processed potato products. Prior to dispersive solid-phase extraction (d-SPE) optimisation, preliminary sample preparation included n-hexane defatting and Carrez clarification to reduce lipid-derived interferences and precipitate suspended starch–protein components present in fried potato matrices. These preliminary clean-up steps substantially improved extract clarity and chromatographic stability before evaluation of different sorbent-based clean-up systems.

Different d-SPE sorbents were subsequently evaluated to optimise extract purification efficiency and analytical performance. The evaluated extraction and clean-up strategies were comparatively assessed based on analytical sensitivity, recovery, repeatability (RSD_r_), and chromatographic performance, and the corresponding results are summarised in Table S1. Considering the overall analytical performance of the evaluated strategies, PSA-based clean-up was selected for subsequent method validation and application. Primary secondary amine (PSA)-based clean-up provided comparatively superior chromatographic selectivity and more stable baseline characteristics than C18-containing systems. The improved performance of PSA was primarily attributed to its ability to remove polar matrix interferences, including organic acids, reducing sugars, and other co-extracted matrix constituents originating from thermally processed potato samples. In contrast, the incorporation of C18 sorbent did not produce substantial improvements in chromatographic selectivity or analytical sensitivity. As shown in Table S1, the incorporation of C18 into the clean-up procedure did not provide any meaningful improvement in overall analytical performance compared with PSA alone. This behaviour was likely associated with the highly polar and water-soluble nature of acrylamide, which limits the effectiveness of non-polar sorbents during extraction and clean-up procedures [27]. Moreover, partial analyte adsorption or signal suppression effects may have occurred under certain experimental conditions when excessive non-polar clean-up was applied. Similarly, the salt-assisted QuEChERS extraction approach involving magnesium sulphate (MgSO_4_) and sodium chloride (NaCl) did not significantly improve extraction efficiency for acrylamide determination, likely because of the high polarity and strong aqueous affinity of the analyte.

The optimised analytical workflow therefore consisted of n-hexane defatting, Carrez clarification, and PSA-based d-SPE prior to HPLC-PDA analysis. Under the selected conditions, cleaner chromatographic profiles, reduced baseline interference, and improved peak symmetry were achieved without requiring derivatisation procedures or extensive purification stages. These findings demonstrated that extensive non-polar clean-up procedures may not necessarily enhance analytical performance for highly water-soluble compounds such as acrylamide and that comparatively simplified clean-up strategies can provide satisfactory analytical reliability for routine acrylamide determination in potato-based food matrices.

### 2.2. Method Validation and Analytical Performance

#### 2.2.1. Linearity

Method linearity was evaluated using seven concentration levels of acrylamide standard solutions ranging from 50 to 2000 µg L^−1^. Each concentration level was analysed in triplicate, and both solvent-based and matrix-matched calibration curves were constructed based on chromatographic peak areas obtained by HPLC-PDA analysis. Good linearity was achieved across the evaluated concentration range, with coefficients of determination (*R*^2^) exceeding 0.99. Matrix effect evaluation, was performed by comparing the slopes of the two calibration curves, indicated slight matrix-induced signal suppression (−19.2%). Quantitative determination of acrylamide was performed using the matrix-matched calibration curve (y = 499x − 18,500 (Figure S1)).

Considering the complexity of thermally processed potato matrices and the relatively low detection wavelength employed (200 nm), this level of matrix effect was considered acceptable. The combined use of n-hexane defatting, Carrez clarification, and PSA-based clean-up effectively minimised matrix-derived interferences, thereby contributing to the satisfactory linearity, recovery, repeatability, and analytical sensitivity achieved by the proposed method.

#### 2.2.2. Sensitivity

Sensitivity was assessed based on the calculated limits of detection (LODs) and limits of quantification (LOQ)s. The LOD and LOQ values determined for acrylamide were 9.7 and 32.3 µg kg^−1^, respectively, indicating satisfactory analytical sensitivity for the determination of low acrylamide concentrations in French fry samples.

The analytical sensitivity achieved in the present study was comparatively evaluated against previously reported methodologies employing different instrumental techniques and sample preparation procedures (Table 1). Despite the comparatively higher sensitivity reported for LC-MS/MS-based methodologies, the proposed HPLC-PDA approach provided satisfactory analytical performance without requiring derivatisation procedures or extensive purification stages. The widespread accessibility and operational simplicity of PDA-based systems additionally enhance the applicability of the proposed method for routine laboratory analysis. No interfering peaks were observed at the retention time of acrylamide in blank matrix chromatograms, confirming satisfactory chromatographic selectivity of the proposed method. Representative HPLC–PDA chromatograms of the acrylamide standard, a French fry sample, and the corresponding standard-spike sample are provided in Figure S2. In addition, comparison of the PDA UV spectra (Figure S3) and peak purity analysis (Figure S4) provided further evidence supporting analyte identification under the proposed chromatographic conditions. Although LC-MS/MS generally provides superior analytical selectivity through mass-based detection and lower detection limits, the proposed HPLC-PDA method provided sufficient chromatographic selectivity for its intended application. This conclusion was further supported by the limited matrix effect (−19.2%), indicating that matrix-derived interferences were effectively controlled following optimisation of the sample preparation procedure. Therefore, despite the relatively low detection wavelength (200 nm), the proposed method enabled reliable quantification of acrylamide in complex French fry matrices and represents a practical and cost-effective alternative for routine laboratories without access to LC-MS/MS instrumentation.

#### 2.2.3. Accuracy and Precision

Accuracy was evaluated through recovery experiments performed using acrylamide-spiked blank matrices at three fortification levels (50, 100, and 500 µg kg^−1^). At the lowest fortification level, selected to verify method performance near the estimated LOQ, a mean recovery of 79.4% and an RSD_r_ of 7.19% were obtained. Recovery values at 100 and 500 µg kg^−1^ were 81.7% and 86.6%, respectively, demonstrating satisfactory extraction efficiency and analytical accuracy. The recovery results further indicated that the proposed extraction and clean-up strategy effectively reduced interfering matrix constituents without adversely affecting acrylamide determination. Furthermore, the obtained recovery values were consistent with the performance criteria established for acrylamide analysis by Commission Regulation (EU) [13], which specifies acceptable recovery ranges between 75% and 110%.

Method precision was assessed from intra-day repeatability studies expressed as relative standard deviation (%RSD). The obtained RSD_r_ values ranged from 3.65% to 7.19%, indicating satisfactory repeatability and instrumental stability. In addition, the obtained repeatability values were substantially lower than the maximum RSD_r_ values estimated according to the modified Horwitz equation and were therefore considered fully compliant with the analytical performance criteria specified in Commission Regulation [13].

### 2.3. Effect of Consecutive Frying Cycles on Acrylamide Formation During Deep-Fat Frying

Frozen French fry samples were subjected to deep-fat frying using sunflower oil, riviera olive oil, and palm oil under repeated frying conditions, and the detected acrylamide concentrations are presented in Table 2. Across all oil types, progressive frying cycles resulted in a pronounced increase in acrylamide formation. In untreated frozen potato samples, acrylamide concentrations increased from 595.7 to 1087.2 µg kg^−1^ in sunflower oil, from 653.9 to 1067.9 µg kg^−1^ in riviera olive oil, and from 405.3 to 704.4 µg kg^−1^ in palm oil between the first and fifth frying cycles. These findings indicate that repetitive thermal exposure substantially altered the frying environment and promoted conditions favourable for acrylamide formation.

The increase in acrylamide concentrations observed during consecutive frying cycles may be associated with progressive thermo-oxidative changes occurring in frying oils during prolonged thermal exposure. Under repetitive frying conditions, oxidative cleavage of unsaturated fatty acids may promote the formation of aldehydes and α,β-unsaturated carbonyl compounds capable of participating in secondary Maillard-type reaction pathways contributing to acrylamide generation [17,36]. In the present study, sunflower oil consistently produced the highest acrylamide concentrations throughout the frying sessions, whereas palm oil exhibited comparatively lower values during the initial cycles.

The observed differences among frying oils may be related, at least in part, to differences in fatty acid composition and oxidative stability. Sunflower oil, characterised by a relatively high content of polyunsaturated fatty acids, has previously been shown to be more susceptible to lipid peroxidation during repeated frying, whereas palm oil generally exhibits greater oxidative stability owing to its higher saturated fatty acid content [37,38]. However, a marked increase in acrylamide concentrations was also observed in palm oil during the later frying sessions, indicating progressive deterioration of oxidative stability under repeated thermal exposure.

Commercial frozen potato products may exhibit acrylamide formation profiles distinct from those of fresh potatoes due to industrial pre-treatments such as blanching, partial frying, and drying. These processes are known to reduce concentrations of free asparagine and reducing sugars, which are the principal precursors involved in acrylamide formation. Kumari et al. [21] reported acrylamide concentrations ranging between 93.1 and 351 µg kg^−1^ in commercially processed frozen potato products, whereas substantially higher concentrations have been observed in fresh potato samples (108–524 µg kg^−1^) subjected to direct frying treatments. Adjija et al. [7] similarly demonstrated that fresh organic potatoes fried in sunflower oil reached acrylamide concentrations of 924.9 µg kg^−1^, while industrially pre-treated frozen potatoes fried in palm oil exhibited considerably lower values (140.4 µg kg^−1^).

A progressive darkening of surface colour was also visually observed during consecutive frying cycles, particularly in sunflower oil samples. Surface browning intensity has frequently been regarded as an indirect indicator of intensified Maillard reaction activity and advanced formation of thermally generated reaction products, including acrylamide. Increased colour development during repeated frying may therefore reflect progressive accumulation of reactive intermediates and enhanced non-enzymatic browning reactions occurring at the food surface [39]. In the present study, acrylamide concentrations obtained during the fourth and fifth frying cycles exceeded the benchmark level of 500 µg kg^−1^ established for French fries, highlighting the potential food safety implications associated with repeated oil use under domestic and commercial frying conditions.

### 2.4. Effect of NaCl Treatment on Acrylamide Mitigation During Deep-Fat Frying

Compared with untreated frozen potato samples, NaCl-treated samples exhibited significantly lower acrylamide concentrations during the initial frying cycles (*p* < 0.05). In sunflower oil, acrylamide concentrations increased from 351.2 to 893.7 µg kg^−1^ between the first and fifth frying sessions, while comparable increasing trends were observed in riviera olive oil and palm oil groups.

Acrylamide reductions of approximately 30–40% were achieved during the early frying cycles following NaCl treatment. The inhibitory effect of NaCl has previously been associated with modifications in water activity, ionic strength, and precursor reactivity within the potato matrix [16,30]. Alterations in localised moisture distribution and ionic interactions may interfere with Maillard-type reaction kinetics, thereby partially limiting the conversion of free asparagine and reducing sugars into acrylamide precursors [30]. Furthermore, NaCl has been suggested to influence thermal reaction equilibria by promoting alternative reaction pathways and reducing the availability of reactive carbonyl intermediates involved in acrylamide generation [15,16].

Despite the initial reduction achieved by NaCl addition, acrylamide concentrations progressively increased during later frying cycles. The gradual loss of the inhibitory effect may be associated with progressive changes in the frying oil during repeated thermal exposure. This phenomenon was particularly evident in sunflower oil samples, where relatively high acrylamide concentrations remained following NaCl treatment. The comparatively high susceptibility of sunflower oil to thermo-oxidative degradation may have enhanced the formation of aldehydic and carbonyl degradation products capable of participating in Strecker-type degradation reactions and secondary acrylamide-forming pathways involving amino–carbonyl interactions [37,38]. The inhibitory effect of NaCl appeared to become progressively less effective as oxidation-derived reaction products accumulated during repetitive frying operations.

**Figure 2:**
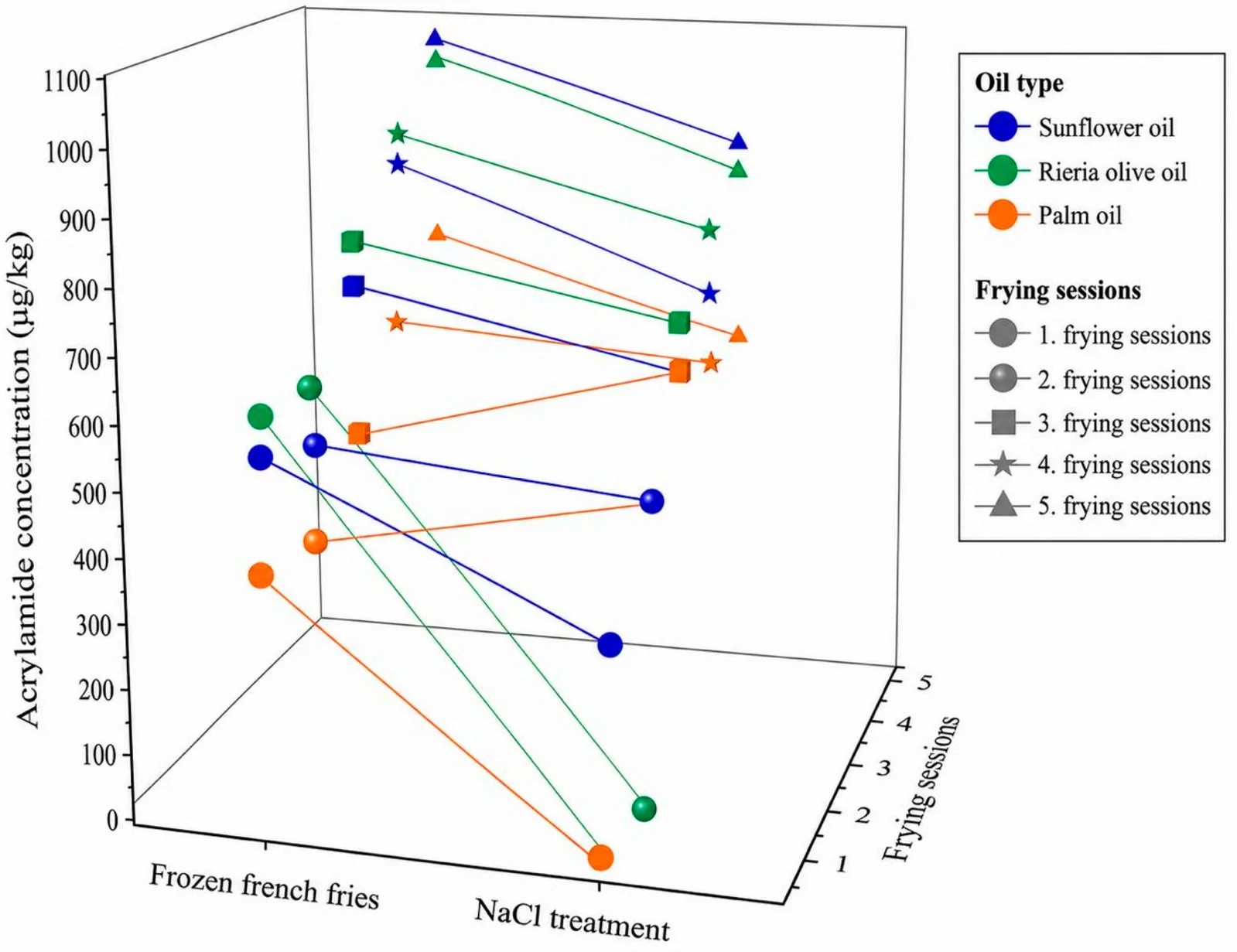
Paired acrylamide concentration profiles of untreated and NaCl-treated frozen French fries across five consecutive frying cycles using sunflower, riviera olive, and palm oils. Different symbols represent the consecutive frying cycles, and lines connect the untreated and NaCl-treated observations obtained under the same frying conditions.

The paired concentration profiles presented in Figure 2 illustrate how the difference in acrylamide concentrations between untreated and NaCl-treated samples varied across the five consecutive frying cycles and among the three frying oils.

Across most frying cycles, acrylamide concentrations were lower in the NaCl-treated samples than in the corresponding untreated samples, although the magnitude of the difference varied according to frying oil and frying cycle. Riviera olive oil and palm oil generally produced lower acrylamide concentrations under NaCl-treated conditions, particularly during the early frying sessions. The comparatively greater oxidative stability of these oils may have contributed to reduced formation of lipid-derived acrylamide precursors, thereby allowing the inhibitory effect of NaCl to remain partially effective.

### 2.5. Comparison of Cooking Methods: Deep-Fat Frying, Oven Baking, and Air Frying

Acrylamide formation in frozen potato products was markedly influenced by cooking method and oil type (Table 3). Among the evaluated treatments, deep-fat frying resulted in significantly higher acrylamide concentrations than oven baking and air frying in most oil systems (*p* < 0.05). In sunflower oil-treated samples, acrylamide concentrations reached 829.6 µg kg^−1^ following deep-fat frying, whereas lower concentrations were observed after oven baking (480.6 µg kg^−1^) and air frying (419.9 µg kg^−1^). Similarly, riviera olive oil produced relatively high acrylamide concentrations under deep-fat frying and air-frying conditions, while palm oil consistently yielded the lowest values among the tested oils.

The comparatively higher acrylamide concentrations observed during deep-fat frying were likely associated with enhanced heat transfer, extensive surface dehydration, and intensified thermo-chemical reactions occurring at the oil-food interface. Direct immersion in hot oil promotes rapid crust formation and creates localised low-moisture microenvironments favourable for Maillard-type amino–carbonyl reactions. In addition, thermal degradation of lipid fractions may contribute additional carbonyl intermediates capable of participating in secondary acrylamide-forming pathways. Comparable observations have previously been reported by Velotto et al. [34], who demonstrated that deep-fat frying formed substantially higher acrylamide concentrations than oven baking and air frying treatments.

Oven baking generally resulted in comparatively lower acrylamide concentrations, particularly in samples treated with riviera olive oil and palm oil, for which concentrations of 289.8 and 285.5 µg kg^−1^ were determined, respectively. This reduction may be associated with slower dehydration kinetics, reduced heat transfer efficiency, and diminished lipid-mediated thermal reactions relative to deep-fat frying. Retention of surface moisture during baking may partially suppress amino–carbonyl interactions by limiting the formation of highly reactive low-water-activity regions at the food surface. However, oil composition remained an important determinant even under reduced-oil cooking conditions, as sunflower oil produced higher acrylamide concentrations than riviera olive oil and palm oil. This trend is consistent with Adjija et al. [7], who reported lower acrylamide formation in frozen potato products baked with palm oil than with sunflower oil.

Air frying also substantially affected acrylamide formation dynamics. Although air fryer systems involve considerably lower oil usage than deep-fat frying, relatively higher acrylamide concentrations were still observed in some treatment groups, particularly in samples treated with riviera olive oil. Shi et al. [10] reported acrylamide concentrations ranging from 93 to 443.9 µg kg^−1^ in frozen potato products subjected to different cooking methods, with air frying and oven baking occasionally producing higher acrylamide concentrations than conventional frying treatments.

The comparatively high acrylamide concentrations observed during air frying appeared to be closely associated with intensified convective heat transfer and accelerated surface dehydration kinetics. High-velocity hot air circulation promotes rapid removal of surface moisture, thereby facilitating the formation of low-water-activity conditions that favour amino–carbonyl condensation reactions and subsequent acrylamide generation. In addition, prolonged exposure to dry thermal conditions may enhance the accumulation of reactive Maillard intermediates within surface crust regions [40]. In the present study, sunflower oil and riviera olive oil samples generally exhibited higher acrylamide concentrations than palm oil-treated samples under air frying conditions, indicating that lipid composition remained an important factor even when oil usage was limited.

The effect of 1% NaCl treatment under air-frying conditions was comparatively limited. Although slight reductions in acrylamide concentrations (2.6–10.1%) were observed, these decreases were not statistically significant (*p* > 0.05). The reduced effectiveness of NaCl may be associated with restricted ion diffusion and rapid moisture removal under intense convective heating conditions. Industrial pre-treatments such as blanching and partial frying may additionally promote the formation of gelatinised starch layers, thereby limiting Na^+^ penetration into potato tissues and reducing the ionic modulation of Maillard-type reaction pathways [40].

**Figure 3:**
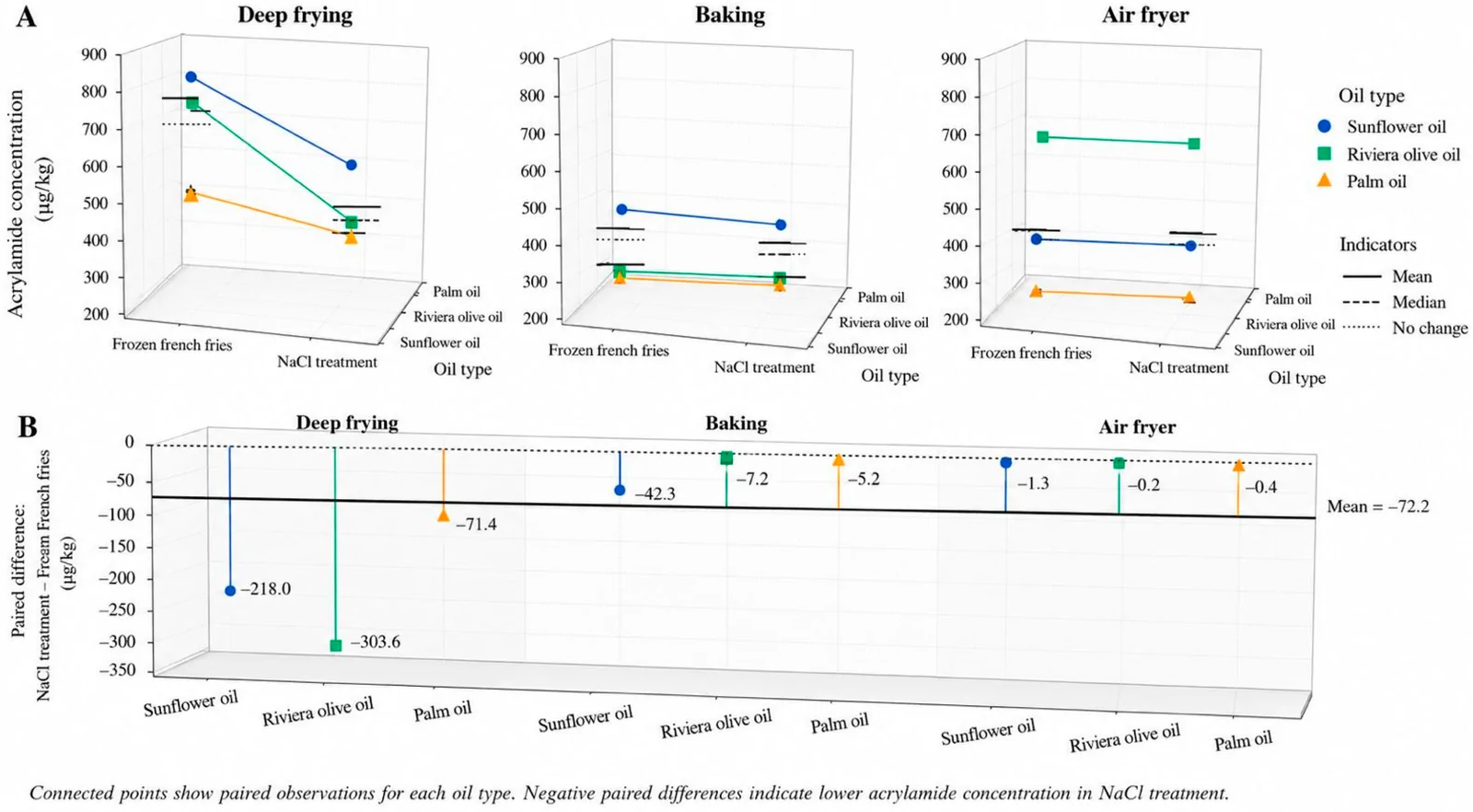
Paired concentration profiles (**A**) and paired difference distributions (**B**) illustrating the effect of NaCl treatment on acrylamide formation across different cooking methods and oil types.

As illustrated in Figure 3, the reduction effect associated with NaCl treatment differed substantially according to processing method and oil type. Deep-fat frying exhibited the largest negative paired concentration shifts, particularly in sunflower and riviera olive oil systems, indicating a stronger reduction tendency under oil-mediated thermal processing conditions. In contrast, baking treatments produced relatively minor paired shifts clustered near the zero reference line, suggesting limited treatment-related variation. Air frying displayed comparatively homogeneous paired distributions across oil systems, reflecting a more consistent but less pronounced reduction response.

Although the present findings provide valuable insights into the influence of thermal processing conditions on acrylamide formation, several limitations should be acknowledged. The experiments were conducted using a single commercially available frozen French fry product and three representative frying oils commonly used in domestic and industrial applications. Therefore, the findings may not fully reflect the variability associated with different potato cultivars, commercial formulations, or frying oils. In addition, physicochemical indicators of oil degradation were not determined, and acrylamide identification relied primarily on retention time and UV detection using HPLC-PDA rather than confirmatory LC-MS/MS analysis. Although the optimised sample preparation procedure, satisfactory chromatographic selectivity, and the limited matrix effect observed supported the suitability of the proposed method for routine acrylamide determination, confirmatory LC-MS/MS analysis together with oil oxidation measurements would provide additional confidence in analyte identification and strengthen mechanistic interpretation in future studies.

## 3. Materials and Methods

### 3.1. Reagents, Chemicals and Standards

HPLC-grade methanol (Chromasolv™) was obtained from Honeywell (Seelze, Germany), while gradient-grade acetonitrile (LiChrosolv^®^) and LC-grade acetone (LiChrosolv^®^) were purchased from Merck KGaA (Darmstadt, Germany). n-hexane was supplied by VWR International (Bois, France). Glacial acetic acid was purchased from Sigma-Aldrich (Steinheim, Germany). Anhydrous MgSO_4_ was obtained from Supelco (Merck KGaA, Darmstadt, Germany), whereas sodium acetate was supplied by Carlo Erba (Val de Reuil, France). PSA sorbent was purchased from Supelco^®^ (Bellefonte, PA, USA), while C18 sorbent was obtained from Agilent Technologies (Santa Clara, CA, USA). NaCl was supplied by Isolab (Eschau, Germany). Zinc sulphate heptahydrate (ZnSO_4_·7H_2_O) and potassium hexacyanoferrate were obtained from Merck KGaA (Darmstadt, Germany). Ultrapure water used throughout the study was produced using a Millipore Direct-Q3 purification system (Millipore, Molsheim, France).

The acrylamide analytical standard (purity ≥ 98%) was purchased from Sigma-Aldrich (St. Louis, MO, USA). A stock standard solution (10 μg mL^−1^) was prepared in ultrapure water and stored at −18 °C until use. Working standard solutions were prepared daily by appropriate dilution of the stock solution with ultrapure water.

### 3.2. Sample Collection and Materials

Commercial frozen French fries used throughout the study were purchased in original 1 kg commercial packages belonging to the same production batch from local supermarkets located in Çorum, Türkiye. Samples were stored at −18 °C until analysis. Sunflower oil and riviera olive oil samples used throughout the study were purchased in multiple original 5 L containers belonging to the same commercial brand and batch number from retail supermarkets located in Çorum, Türkiye. Palm oil was obtained in original 18 L metal containers from an international retail chain located in Ankara, Türkiye.

Frozen French fries were divided into two experimental groups prior to thermal processing. The first group consisted of untreated frozen samples, whereas the second group was subjected to a 1% NaCl pre-treatment prior to thermal processing.

### 3.3. Cooking Procedures

Different thermal processing techniques, including deep-fat frying, oven baking, and air frying, were comparatively evaluated. Sunflower oil, riviera olive oil, and palm oil were selected as representative frying oils commonly used in domestic and industrial applications. Prior to thermal processing, acrylamide concentrations in frozen French fry samples were determined. Following thermal processing, samples were allowed to cool to room temperature under ambient conditions, homogenised using a laboratory blender, and stored at 4 °C for a maximum of 48 h prior to analysis.

Deep-fat frying experiments were carried out using an industrial fryer (Bilgeinox, Inoksan, İstanbul, Türkiye). Portions of approximately 250 g of frozen potato samples were fried in 2 L of vegetable oil (sunflower oil, riviera olive oil, or palm oil) at 190 °C until a characteristic golden-yellow colour was achieved. Frying time was standardised at 2.0 ± 0.1 min for all oil types. To evaluate the effect of repeated oil use, frying procedures were repeated over five consecutive frying cycles. For each frying cycle, a fresh 250 g portion of frozen French fries from the same production batch was used to eliminate any potential influence of repeated heating of the potato material itself. The frying oil remained continuously heated at 190 °C throughout the consecutive frying cycles in order to simulate typical commercial frying conditions. The interval between successive frying cycles was limited to approximately 2 min, reflecting the time required to remove the fried sample and load the subsequent potato portion. Following frying, samples were homogenised and stored at 4 °C until analysis.

For oven-baking experiments, approximately 250 g of potato samples were evenly distributed onto baking trays lined with parchment paper and lightly coated with 10 mL of vegetable oil according to the experimental design. Baking was performed in a domestic electric oven (Simfer, Istanbul, Türkiye) at 190 °C for 30 min.

Air-frying experiments were conducted using an air fryer device (FitFry XL, Beko, Istanbul, Türkiye). Approximately 250 g of potato samples was placed into the cooking basket either without oil addition or after the application of 10 mL of vegetable oil, depending on the experimental conditions. Cooking was carried out at 200 °C for 20 min.

### 3.4. Sample Preparation

Acrylamide extraction was performed using a modified QuEChERS-based procedure adapted from previous studies [19,24]. During method optimisation, different clean-up strategies involving MgSO_4_ + PSA, MgSO_4_ + PSA + C18, and salt-assisted QuEChERS extraction (MgSO_4_ + NaCl) were comparatively evaluated to assess their influence on extraction recovery and chromatographic performance. Based on the obtained analytical results, the optimized extraction and clean-up procedure was established as follows.

Briefly, 2.5 g of homogenised sample was transferred into a 50 mL polypropylene centrifuge tube, followed by the addition of 10 mL of ultrapure water. For defatting purposes, 5 mL of n-hexane was added, and the mixture was shaken on a programmable rotator (Multi RS-60 rotator, Biosan, Riga, Latvia) at 250 rpm for 20 min. The samples were subsequently centrifuged at 4000 rpm for 5 min (DLab, Beijing, China), after which the upper organic layer was discarded.

To facilitate protein precipitation and matrix clarification, 1 mL each of Carrez I solution (15 g/100 mL potassium hexacyanoferrate (II)) and Carrez II solution (30 g/100 mL zinc sulfate heptahydrate) was added to the extracts. The mixture was vortexed for 30 s and centrifuged at 4000 rpm for 5 min. Following centrifugation, three distinct phases consisting of an upper particulate layer, an intermediate aqueous layer containing acrylamide, and a lower sediment phase were obtained.

The intermediate aqueous phase was carefully transferred into a separate centrifuge tube, and an additional 1 mL each of Carrez I and Carrez II solutions was subsequently added to further improve matrix clarification. After vortex mixing for 30 s, the samples were centrifuged again at 4000 rpm for 5 min. The purified aqueous phase was then subjected to d-SPE clean-up using 150 mg MgSO_4_ and 50 mg PSA sorbent. The mixture was vortexed for 30 s and centrifuged at 4000 rpm for 5 min. Finally, the extract was filtered through a 0.45 μm membrane filter, transferred into LC vials, and analysed using HPLC-PDA.

### 3.5. HPLC-PDA Analysis

Qualitative and quantitative analyses of acrylamide were carried out using a Shimadzu HPLC system equipped with a Shimadzu SPD-M20A photodiode array (PDA) detector (Shimadzu, Tokyo, Japan). Chromatographic separation was achieved using an Inertsil^®^ ODS-3 reversed-phase column (250 × 4.6 mm, 5 μm; GL Sciences, Tokyo, Japan). The mobile phase consisted of methanol-water (10:90, *v*/*v*) and was delivered under isocratic conditions at a flow rate of 0.5 mL min^−1^. The injection volume was 20 μL, while the column temperature was maintained at 30 °C. Detection was performed at a wavelength of 200 nm.

### 3.6. Method Validation

Method validation was performed to evaluate the analytical performance of the proposed method in terms of linearity, matrix effect, sensitivity, recovery, and repeatability. Calibration curves were constructed using seven concentration levels of acrylamide standard solutions (50, 100, 250, 500, 1000, 1500, and 2000 µg L^−1^). Each concentration level was analysed in triplicate, and linear regression analysis was carried out based on peak area responses.

To evaluate potential matrix-induced effects on detector response, matrix effect was assessed by comparing solvent-based and matrix-matched calibration curves prepared at identical concentration levels (50–2000 µg L^−1^). Matrix-matched calibration standards were prepared by spiking frozen French fry extracts following sample preparation. Matrix effect was calculated using Equation (1):

(1) $$ME\;\left(\%\right)=\left(\frac{{slope}_{matrix}}{{slope}_{solvent}}-1\right)\times 100$$

Considering the observed matrix-induced signal suppression, quantitative determination of acrylamide in all samples was performed using the matrix-matched calibration curve.

Recovery studies were conducted by spiking homogenised blank frozen French fries with acrylamide at concentration levels of 50, 100 and 500 µg kg^−1^ (*n* = 8). Spiked samples were allowed to equilibrate overnight prior to extraction and analysis according to the proposed method. Repeatability was expressed as %RSD values obtained from recovery experiments. The LOD and LOQ values were estimated based on the standard deviation (SD) of replicate spiked samples analysed at the 50 µg kg^−1^ fortification level. LOD and LOQ values were calculated as three and ten times the SD, respectively. The 50 µg kg^−1^ fortification level was selected because it is close to the estimated LOQ, allowing experimental verification of method performance, including recovery and precision, in the low concentration range.

### 3.7. Statistical Analysis

All experiments were performed in triplicate. Prior to analysis of variance (ANOVA), normality and homogeneity of variance assumptions were verified using the Shapiro–Wilk and Levene tests, respectively. Statistical differences among acrylamide concentrations obtained under different cooking techniques and vegetable oil treatments were evaluated using one-way ANOVA followed by Duncan’s multiple comparison test. Statistical analyses were performed using IBM SPSS statistics version 27 (IBM Corp., Armonk, NY, USA). The five frying cycles were sequential observations obtained from the same continuously heated frying process and were not considered independent experimental replicates.

## 4. Conclusions

In the present study, a modified QuEChERS-based extraction and clean-up strategy coupled with HPLC-PDA analysis was successfully developed and optimised for the determination of acrylamide in French fries. The proposed analytical approach provided satisfactory linearity, sensitivity, recovery, repeatability, and chromatographic selectivity for acrylamide analysis in thermally processed potato matrices. The combined use of n-hexane defatting, Carrez clarification, and PSA-based d-SPE enabled efficient reduction in matrix interferences without requiring derivatisation procedures or extensive purification steps. The optimised analytical method was subsequently applied to evaluate the influence of different thermal processing conditions on acrylamide formation in frozen French fries. Deep-fat frying formed substantially higher acrylamide concentrations than oven baking and air frying, highlighting the important roles of heat transfer efficiency, dehydration kinetics, and lipid-mediated thermal reactions in acrylamide formation. Repeated frying cycles markedly increased acrylamide concentrations, particularly in sunflower oil systems. In contrast, palm oil generally exhibited comparatively lower acrylamide formation during the initial frying cycles, likely due to its greater oxidative stability. NaCl pre-treatment partially reduced acrylamide formation during early frying operations; however, its inhibitory effect progressively decreased under repetitive thermal exposure and intensified oil degradation conditions. The findings demonstrated that both thermal processing technology and frying oil composition substantially influenced acrylamide formation in potato-based foods. The proposed analytical methodology offers a comparatively simplified, cost-effective, and analytically reliable approach for routine acrylamide determination in starch-rich food matrices while also providing mechanistic insights into processing-dependent chemical transformations associated with acrylamide generation during thermal processing.

## Figures and Tables

**Table 1:** Comparison of analytical methods for acrylamide determination in potato-based matrices.

Analytical Technique	Extraction and Clean-Up Procedure	LOD (µg kg^−1^)	LOQ (µg kg^−1^)	Ref.
LC-MS/MS	n-hexane defatting followed by purification using Oasis HLB SPE and Bond Elut AccuCAT SPE cartridges	10	–*	[10]
LC-MS/MS	SPE purification	–*	5	[29]
LC-MS/MS	Modified QuEChERS extraction employing MgSO_4_/NaCl salts and dispersive SPE clean-up (50 mg PSA and 150 mg MgSO_4_)	6.1	18.6	[7]
LC-MS/MS	Sequential purification using Oasis HLB and Bond Elut AccuCAT cartridges	–*	5	[30]
LC-MS/MS	QuEChERS salt extraction (MgSO_4_ + NaCl)	4.8	18.2	[20]
LC-MS/MS	QuEChERS salt extraction (PSA + MgSO_4_ + C18)	0.7	2.0	[24]
LC-MS/MS	SPE purification	10.3	30.9	[31]
LC-MS/MS	Extraction using acetic acid/water and ethyl acetate followed by Carrez I and Carrez II clarification	10	15	[32]
LC-MS/MS	SPE purification (Oasis HLB cartridges)	0.23	0.77	[33]
GC-MS	MSPD extraction using C18 sorbent followed by derivatisation to 2,3-dibromopropionamide (2,3-DBPA) using KBr, HBr, and sodium thiosulfate	14	42	[34]
GC-MS/MS	SPE purification using isolute multimode and isolute ENV+ cartridges combined with bromination derivatisation	6.9	20.8	[15]
GC-MS/MS	SPE purification using isolute multimode and isolute ENV+ cartridges followed by derivatisation to 2,3-dibromopropionamide (2,3-DBPA) using KBr, HBr, and bromine water	10.3	30.9	[16]
HPLC-PDA	Hexane defatting combined with Carrez I and Carrez II precipitation	80	150	[35]
HPLC-PDA	n-hexane defatting followed by Carrez I and Carrez II clarification and dispersive SPE clean-up (50 mg PSA and 300 mg MgSO_4_)	9.7	32.3	Present study

LOD: limit of detection; LOQ: limit of quantification; SPE: solid-phase extraction; MSPD: matrix solid-phase dispersion; PSA: primary secondary amine; HPLC-PDA: high-performance liquid chromatography coupled with photodiode array detection; LC-MS/MS: liquid chromatography-tandem mass spectrometry; GC-MS/MS: gas chromatography-tandem mass spectrometry. –*: Only LOD or LOQ values were reported in the original publication.

**Table 2:** Effect of consecutive frying cycles and frying oil type on acrylamide formation in frozen French fries.

	Acrylamide Concentration (µg kg^−1^)		
Frying Cycle	Sunflower Oil	Riviera Olive Oil	Palm Oil
1	595.7 ± 14.2 ^b,B^	653.9 ± 12.3 ^a,C^	405.3 ± 9.2 ^a,A^
2	547.2 ± 10.3 ^a,B^	636.5 ± 9.2 ^a,C^	387.9 ± 14.9 ^a,A^
3	733.7 ± 23.3 ^c,B^	807.6 ± 27.5 ^b,C^	501.6 ± 11.0 ^b,A^
4	906.5 ± 17.2 ^d,B^	954.3 ± 20.2 ^c,C^	625.1 ± 21.2 ^c,A^
5	1087.2 ± 29.6 ^e,B^	1067.9 ± 18.8 ^d,B^	704.4 ± 29.5 ^d,A^

Values are expressed as mean ± standard deviation (*n* = 3). ^a–e^ Different lowercase letters within the same column indicate significant differences between frying cycles (*p* < 0.05). ^A–C^ Different uppercase letters within the same row indicate significant differences among frying oils (*p* < 0.05).

**Table 3:** Effect of cooking method and oil type on acrylamide formation in frozen French fries.

	Acrylamide Concentration (µg kg^−1^)		
Vegetable Oil Type	Deep-Fat Frying	Baking	Air fryer
Sunflower oil	829.6 ± 40.6 ^b,C^	480.6 ± 15.1 ^b,B^	419.9 ± 20.8 ^b,A^
Riviera olive oil	776.1 ± 17.0 ^b,C^	289.6 ± 7.7 ^a,A^	673.6 ± 16.8 ^c,B^
Palm oil	526.7 ± 40.6 ^a,B^	285.5 ± 28.1 ^a,A^	272.1 ± 29.5 ^a,A^

Values are expressed as mean ± standard deviation (*n* = 3). ^a–c^ Different lowercase letters within the same column indicate significant differences among oil types (*p* < 0.05). ^A–C^ Different uppercase letters within the same row indicate significant differences among cooking methods (*p* < 0.05).

## Data Availability

The original contributions presented in the study are included in the article and Supplementary Materials, further inquiries can be directed to the corresponding author.

## Supplementary Materials

The following supporting information can be downloaded at: https://www.mdpi.com/article/10.3390/molecules31162777/s1, Table S1: Comparative analytical performance of the evaluated extraction and clean-up strategies used during optimisation of the QuEChERS procedure for acrylamide determination; Figure S1: Matrix-matched calibration curve used for the quantitative determination of acrylamide by HPLC-PDA; Figure S2: Representative HPLC-PDA chromatograms of (A) acrylamide standard (250 µg kg^−1^), (B) a French fry sample containing acrylamide (sample A), and (C) the same sample after spiking with acrylamide standard; Figure S3: PDA UV spectra of (A) acrylamide standard (250 µg kg^−1^), (B) a French fry sample containing acrylamide (sample A), and (C) the same sample after spiking with acrylamide standard; Figure S4: PDA peak purity analysis demonstrating the spectral homogeneity of the acrylamide peak in (A) a French fry sample containing acrylamide (sample A) and (B) the same sample after spiking.
