# Supplementary material for: Processing-Dependent Acrylamide Formation in French Fries: Optimisation of QuEChERS-Based Extraction and HPLC-PDA Determination
Source: Molecules. 2026 Aug 10;31(16):2777. doi: 10.3390/molecules31162777 (PMC13515462; doi:10.3390/molecules31162777)
Supplement: Supplementary file 1 [file molecules-31-02777-s001.zip › molecules-4369041-supplementary.pdf]

Supplementary Material

Processing-dependent acrylamide formation in French fries: Optimisation of QuEChERS-based extraction and HPLC-PDA determination

Nimo Hussein Yussuf [1,2], Eylem Odabas [2], Fatma Oznur Afacan [3] and Bulent Kabak [2,*]

[1] Department of Nutrition and Food Science, Edna Adan University, Iftin Road, Hargeisa, Somaliland.
[2] Department of Food Engineering, Faculty of Engineering and Natural Sciences, Hitit University, Corum, 19030, Türkiye
[3] Department of Nutrition and Dietetics, Hamidiye Faculty of Health Sciences, University of Health Sciences, Istanbul, 34668, Türkiye
* Correspondence: bulentkabak@hitit.edu.tr (B.K.)

Table S1. Comparative analytical performance of the evaluated extraction and clean-up strategies used during optimisation of the QuEChERS procedure for acrylamide determination.

**Clean-up strategy**	**LOD (μg kg⁻¹)**	**LOQ (μg kg⁻¹)**	**Recovery (%)**			**RSD_r (%)**			**Observation**
			**50 μg kg⁻¹**	**100 μg kg⁻¹**	**500 μg kg⁻¹**	**50 μg kg⁻¹**	**100 μg kg⁻¹**	**500 μg kg⁻¹**	
$MgSO_4$ +NaCl	12.8	42.7	72.7	71.5	72.8	8.42	3.97	6.13	Lower analytical recovery and reduced overall analytical performance
$MgSO_4$ + PSA*	9.7	32.3	79.4	81.7	86.6	7.19	4.82	3.65	Selected as the final clean-up strategy because of the most favourable overall analytical performance
$MgSO_4$ + PSA + C18	10.2	34.0	76.5	81.2	85.4	5.04	5.12	2.78	Comparable recovery to PSA but no additional improvement in analytical performance

* The PSA-based clean-up strategy was selected for subsequent validation and application because it provided the most favourable overall analytical performance, considering analytical sensitivity (LOD and LOQ), recovery, precision (RSDr), and chromatographic performance.

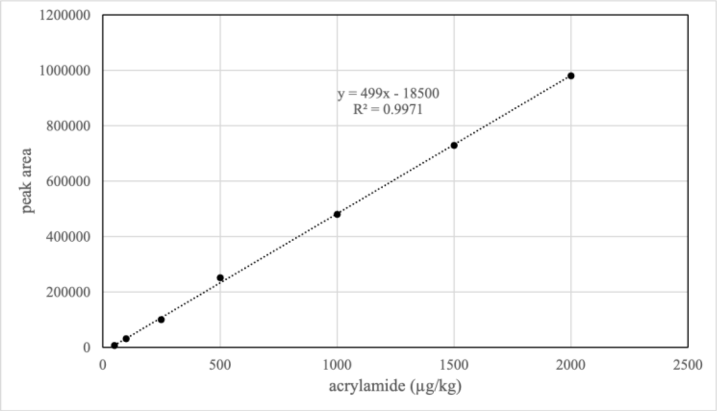

Figure S1. Matrix-matched calibration curve used for the quantitative determination of acrylamide by HPLC-PDA.

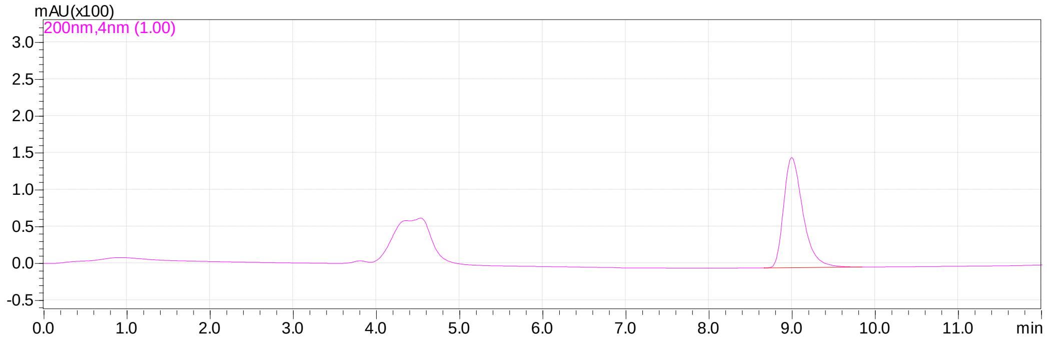

A) 250 μg kg⁻¹ acrylamide standard solution

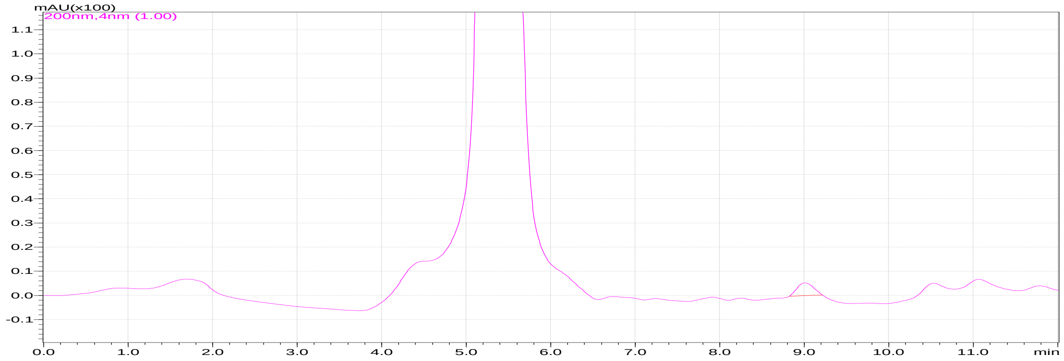

B) French fries sample containing acrylamide (Sample A)

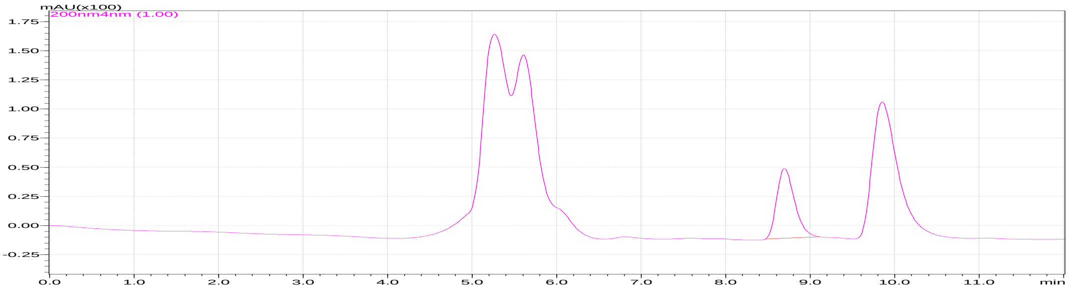

C) Sample A + acrylamide standard

Figure S2. Representative HPLC-PDA chromatograms of (A) acrylamide standard (250 μg kg⁻¹), (B) a French fry sample containing acrylamide (Sample A), and (C) the same sample after spiking with acrylamide standard.

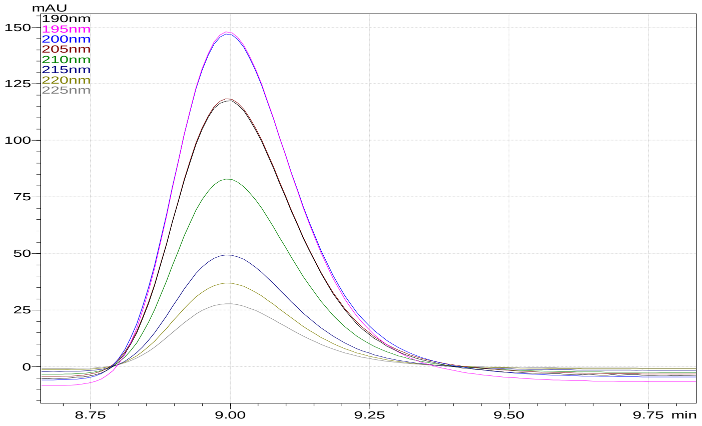

A) 250 µg kg⁻¹ acrylamide standard solution

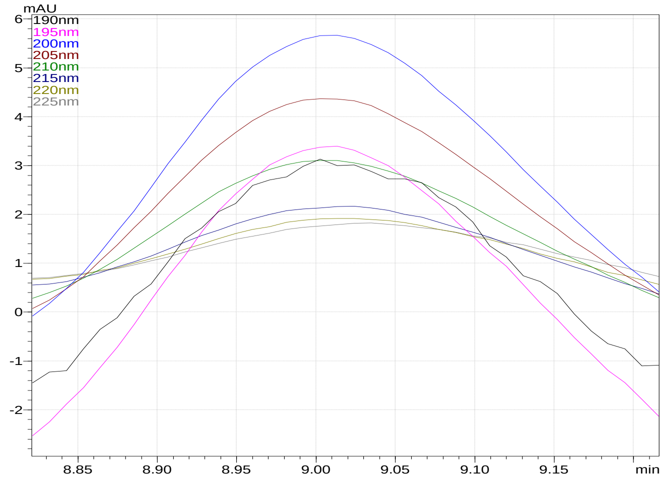

B) French fries sample (Sample A)

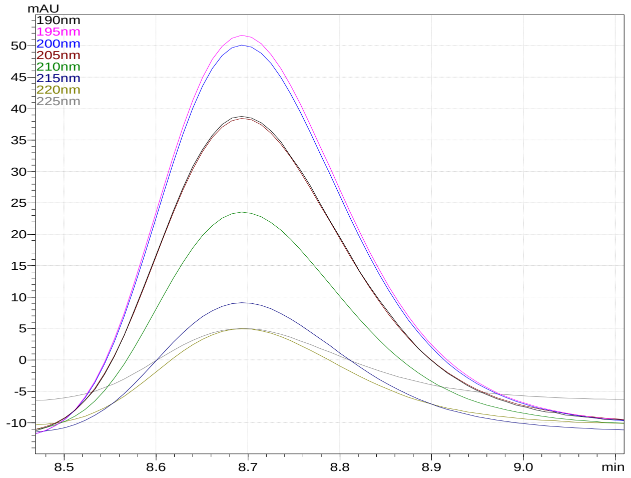

C) Sample A + acrylamide standard

Fig S3. PDA UV spectra of (A) acrylamide standard (250 µg kg⁻¹), (B) a French fry sample containing acrylamide (Sample A), and (C) the same sample after spiking with acrylamide standard.

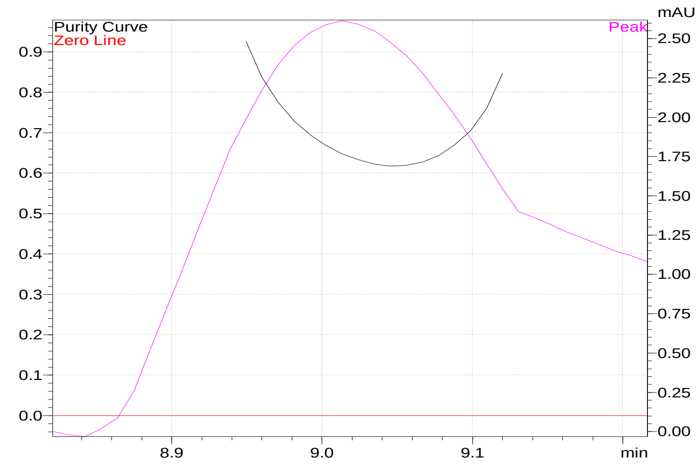

A) French fries sample (Sample A)

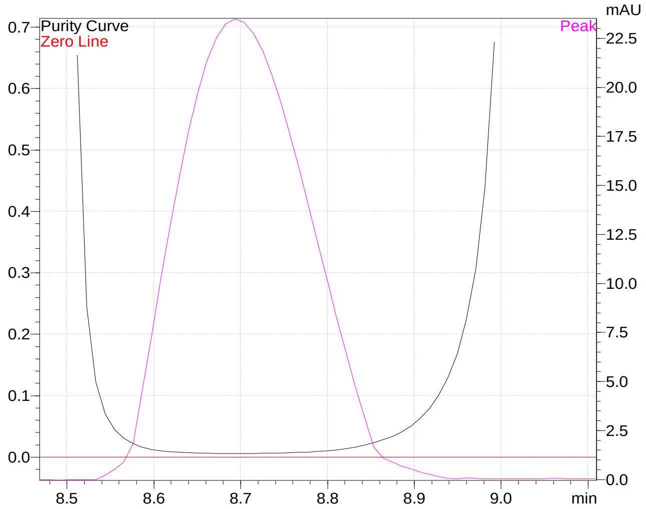

B) Sample A + acrylamide standard

Figure S4. PDA peak purity analysis demonstrating the spectral homogeneity of the acrylamide peak in (A) a French fry sample containing acrylamide (Sample A) and (B) the same sample after spiking with acrylamide standard.
